# E-Cadherin (*CDH1* Gene) Germline Mutations in Gastric Cancer: Evolutions and Innovations

**DOI:** 10.3390/cancers12102920

**Published:** 2020-10-11

**Authors:** Giovanni Corso, Bernardo Bonanni

**Affiliations:** 1 Division of Breast Surgery, European Institute of Oncology, IRCCS, 20141 Milan, Italy; 2 Department of Oncology and Hemato-Oncology, University of Milan, 20141 Milan, Italy; 3 Division of Cancer Prevention and Genetics, European Institute of Oncology, IRCCS, 20141 Milan, Italy; bernardo.bonanni@ieo.it

## 1. Introduction

Family history has contributed greatly to understanding inherited diseases throughout the centuries, in particular familial and hereditary cancer syndromes. To assess the cancer risk for unaffected members and to identify a possible genetic cause, it is important to describe a detailed family history, including information about life status, gender, age at onset, affected members and the number of generations.

Therefore, a careful anamnesis focused on oncological data could lead to the diagnosis of familial and/or hereditary cancer. The definition of “familial” is a non-specific status and indicates only a positive family history. Rather, the diagnosis of hereditary cancer has to be confirmed by some specific genetic tests. 

In 1964, Jones identified a Māori family with an exceptional frequency of gastric tumors: in a pedigree with 98 members, 28 were affected by primary gastric carcinoma, and within a period of 30 years, over 25 family members died from this disease [1]. Many years later, in 1998, Guilford et al. first identified three different *CDH1* germline mutations in three Māori kindred from New Zealand with a strong familial cluster of diffuse gastric cancer (DGC). These Authors described a splice site (G1008T), a frameshift (2382–2386, C ins), and a premature termination (TAG, C2095T) germline mutation in the E-cadherin gene [2], respectively.

On the basis of clinical criteria, subsequently in 1999, the first International Gastric Cancer Linkage Consortium (IGCLC) defined families with the Hereditary Diffuse Gastric Cancer (HDGC) syndrome associated with *CDH1* germline mutations as those fulfilling one of the following features [3]: (a) two or more documented cases of diffuse gastric cancer in first- or second-degree relatives, with at least one diagnosed before the age of 50; (b) three or more cases of documented diffuse gastric cancer in first- or second-degree relatives, independent of the age of onset.

However, due to the increase in the *CDH1* germline mutation rate, those initial criteria have become insufficient. 

## 2. Current Position

To date, it is assessed that about 80–90% of GC appears as sporadic form, 10–20% are within a familial setting, and only 1–3% are related to documented germline mutations. Specifically, for HDGC syndrome related to *CDH1* germline mutations, the cumulative incidence of GC at age 80 years is about 70% for males and 56% for females. An increased risk of lobular breast cancer (BC) is also documented [4]. In this case, the risk of BC for females is about 42% [5]. 

Recently, novel international guidelines for *CDH1* genetic screening have been published [6] as follows:

### 2.1. Family Criteria

(a) ≥2 cases of gastric cancer in family regardless of age, with at least one DGC;
(b) ≥1 case of DGC at any age and ≥1 case of lobular BC at age <70 years in different family members;
(c) ≥2 cases of lobular BC in family members <50 years of age.

### 2.2. Individual Criteria

(d) DGC at age <50 years;
(e) DGC at any age in individuals of Māori ethnicity;
(f) DGC at any age in individuals with a personal or family history (first-degree relative) of cleft lip or cleft palate;
(g) History of DGC and lobular breast cancer, both diagnosed at age <70 years;
(h) Bilateral lobular BC, diagnosed at age <70 years;
(i) Gastric in situ signet ring cells or pagetoid spread of signet ring cells in individuals <50 years of age.

Prophylactic total gastrectomy is the only life-saving option in *CDH1* asymptomatic mutation carriers fulfilling the abovementioned criteria, but probably not indicated in *CDH1* incidental findings without a clear family history of GC [7].

**Figure 1:**
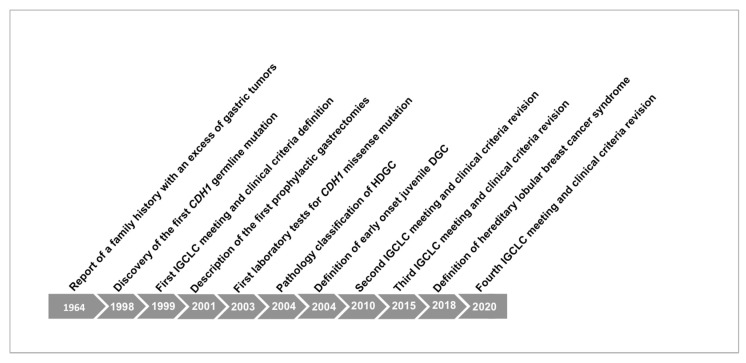
Historical overview of Herditary Diffuse Gastric Cancer (HDGC) syndrome and *CDH1* germline mutation.

Figure 1 shows briefly the historical overview of HDGC and the main discoveries since its early identification.

## 3. Future Perspectives

With the widespread introduction of MultiGene Panel Testing (MGPT) in clinical practice, we are observing an increased rate of *CDH1* germline mutations in apparently healthy individuals and without any correlation with the classic HDGC syndrome [8]. The identification of unexpected *CDH1* germline mutations in the absence of specific clinical criteria suggests that HDGC syndrome may be a more complex syndrome than the one originally defined [9]. A cross-sectional prevalence study from the University of Southern California, Los Angeles, included all patients who underwent MGPT between 2012–2014. A total of 27,254 individuals were identified, and 20 (0.07%) of these patients were selected as having a *CDH1* mutation. However, if we considered only the clinic cohort, four (1.26%) of 318 had a pathogenic *CDH1* mutation [8].

The unquestionable direction for the management of this complex inherited cancer predisposition syndrome is the multidisciplinary approach in high-specialized cancer centers. Opinions of pathologists, surgeons, biologists, geneticists, epidemiologists, and radiologists are required equally for a correct approach to the HDGC syndrome. Further, considering the possible impacts of the HDGC syndrome on the individuals and their lives, the psychological aspects should be taken into consideration [10,11,12].
